# Machine learning-based screening of asthma biomarkers and related immune infiltration

**DOI:** 10.3389/falgy.2025.1506608

**Published:** 2025-01-29

**Authors:** Xiaoying Zhong, Jingjing Song, Changyu Lei, Xiaoming Wang, Yufei Wang, Jiahui Yu, Wei Dai, Xinyi Xu, Junwen Fan, Xiaodong Xia, Weixi Zhang

**Affiliations:** ^1^ Allergy and Clinical Immunology Center, The Second Affiliated Hospital and Yuying Children’s Hospital of Wenzhou Medical University, Wenzhou, Zhejiang, China; ^2^ Department of Pediatric Allergy and Immunology, The Second Affiliated Hospital and Yuying Children’s Hospital of Wenzhou Medical University, Wenzhou, Zhejiang, China; ^3^ The 2nd Ward of Pediatrics, Jinhua Maternal and Child Health Care Hospital, Jinhua, Zhejiang, China; ^4^ Renji College, Wenzhou Medical University, Wenzhou, Zhejiang, China

**Keywords:** asthma, differentially expressed genes, machine learning, support vector machine recursive feature elimination (SVM-RFE), least absolute shrinkage and selection operator (LASSO) regression model

## Abstract

**Introduction:**

Asthma has an annual increasing morbidity rate and imposes a heavy social burden on public healthcare systems. The aim of this study was to use machine learning to identify asthma-specific genes for the prediction and diagnosis of asthma.

**Methods:**

Differentially expressed genes (DEGs) related to asthma were identified by examining public sequencing data from the Gene Expression Omnibus, coupled with the support vector machine recursive feature elimination and least absolute shrinkage and selection operator regression model. Gene Ontology (GO), Kyoto Encyclopedia of Genes and Genomes (KEGG), Gene set enrichment analysis and correlation analyses between gene and immune cell levels were performed. An ovalbumin-induced asthma mouse model was established, and eukaryotic reference transcriptome high-throughput sequencing was performed to identify genes expressed in mouse lung tissues.

**Results:**

Thirteen specific asthma genes were obtained from our dataset analysis (*LOC100132287*, *CEACAM5*, *PRR4*, *CPA3*, *POSTN*, *LYPD2*, *TCN1*, *SCGB3A1*, *NOS2*, *CLCA1*, *TPSAB1*, *CST1*, and *C7orf26*). The GO analysis demonstrated that DEGs linked to asthma were primarily related to positive regulation of guanylate cyclase activity, gpi anchor binding, peptidase activity and arginine binding. The renin-angiotensin system, arginine biosynthesis and arginine and proline metabolism were the key KEGG pathways of DEGs. Additionally, the genes *CEACAM5*, *PRR4*, *CPA3*, *POSTN*, *CLCA1*, and *CST1* expression levels were positively associated with plasma cells and resting mast cells. The mouse model revealed elevated *nos2* and *clca1* expression in the asthmatic mouse group compared with that in normal mice, which was consistent with the findings in asthmatic patients.

**Discussion:**

This study identified new marker genes for the prediction and diagnosis of asthma, which can be further validated and applied clinically

## 1 Introduction

Asthma is a chronic respiratory illness marked by inflammation and remodeling of the airways that is triggered by complex genetic regulation and environmental exposure to allergens (1). It is caused by swelling and increased mucus in the airways (inflammation) and narrowing of the airways due to muscle spasm (bronchospasm) leading to bronchial overreaction and airway obstruction (2). Asthma affects 5%–10% of the population in many developed countries. In China, approximately 4.2% of adults over the age of 20 years and older have asthma. There are more than 300 million people with asthma worldwide, and its prevalence is increasing every year (3). Asthma usually occurs in preschool years and causes reducing quality of life and early death, which leads to a large public health burden.

Many patients with asthma use self-care and pharmacological therapies to control their symptoms. Recently, monoclonal antibodies have been applied to benefit patients (4). However, their efficacy differs owing to the diverse forms of asthma, and some individuals do not respond to current asthma treatments (5). In recognition of inadequacies in the current understanding of asthma mechanisms, our research highlights the need of having a thorough grasp of diagnosis and immunological variability in asthma.

Asthma involves multiple inflammatory responses. The initial barrier to resistance against microorganisms, gases, and allergens is the bronchial epithelial cells, which is also the centre of the inflammatory response (6). First, allergens can be eliminated by airway epithelial cells via mucus (7). Second, by using pattern recognition receptors, airway epithelial cells may identify chemicals associated with pathogens or hazards, and release cytokines and chemokines (such as IL25, TSLP, CCL5, and CCL22), activate dendritic cells, connect innate and adaptive immunity, and trigger local immune responses (8, 9). Third, they function as antigen-presenting cells, which help trigger type II immunological responses by causing naive T cells to differentiate into CD4+ T cells (10). Therefore, we chose samples of bronchial epithelial cells from public datasets for sequencing and analysis in this study.

Machine learning is a collection of computational intelligence techniques that allows a computer to learn a task autonomously, improving its experience without being explicitly programmed. This method can be used to analyze large amounts of data, establish complex and nonlinear relationships, and identify patterns and relationships between data and interesting outcomes. Zhang et al. identified the immune infiltration-related diagnostic genes *SLC27A3* and *STAU1* using machine learning in patients with Chronic Obstructive Pulmonary Disease. The area under the receiver operating characteristic (ROC) curve (AUC) was up to 0.900 and 0.971, which demonstrated their high diagnostic value (11). Potential biomarkers of idiopathic pulmonary frailty were previously identified using the least absolute shrinkage and selection operator (LASSO) regression model, random forest algorithms, and support vector machine recursive feature elimination (SVM-RFE). These methods were very helpful in enabling an early diagnosis and enhancing prognosis (12). Research to date shows that machine learning helps uncover the potential causal mechanisms of asthma with good predictive efficacy and can generate new hypotheses (13, 14).

Despite its significant impact on public health, the diagnosis of asthma mostly depends on symptoms and accompanying testing, which does not allow for prediction and treatment. Our study explored differentially expressed genes (DEGs) in asthma by analyzing the bronchial epithelial cell transcriptome of the GSE63142 and GSE158752 databases from the Gene Expression Omnibus (GEO) public sequencing data using machine learning approaches. We evaluated the diagnostic utility of these genes using the AUC after doing functional, pathway, and gene set enrichment analyses of the DEGs. We hope to contribute to the genetic diagnosis and prediction of asthma in the future.

## 2 Methods

### 2.1 Microarray data processing and analysis

We obtained the GSE63142 and GSE158752 asthma datasets from the GEO database of the National Center for Biotechnology Information (NCBI) (https://www.ncbi.nlm.nih.gov/geo/). The GSE63142 dataset (GPL6480 platform) (15) was uploaded in 2014 and included transcriptome studies of the bronchial epithelial cells from 27 healthy participants and 128 patients with asthma. In the GSE158752 dataset (GPL18573 platform) (16), 50 bronchial epithelial cell samples from patients with asthma and 17 normal samples were obtained. R software (version 4.2.2; https://www.r-project.org/) and RStudio software (version 4.2.2; https://www.r-project.org/) were used to process and analyze the datasets.

### 2.2 Analysis of differentially expressed genes

All profiles of the gene transcription microarray data were pre-processed utilising the “SVA” package (version 3.46.0), which included background correction and normalization. Gene symbols were annotated based on the annotation data. To execute advanced feature selection and visualization, we utilized GSE63142 as a training set for the DEGs analysis and used GSE158752 as a test set. We examined transcriptome samples from healthy controls and asthma patients to support the expression of the crucial genes identified. The GSE158752 dataset was used for verifying the core gene differential expression. Using the “limma” R package, we determined the DEGs between samples from patients with asthma and healthy subjects using a conservative threshold (|log2FC| > 1.0, *p* < 0.05). We utilised the “pheatmap” (version 1.0.12) and “ggplot2” packages (version 3.4.2) to generate a heatmap and volcano plot, respectively.

### 2.3 Function, pathway and gene set enrichment analysis and protein–protein interaction network analysis of DEGs

Gene Ontology (GO), Kyoto Encyclopedia of Genes Genomes (KEGG) pathway enrichment analyses were used to identify the characteristic biological, molecular, and cellular attributes and reveal associated enriched pathways. Gene Set Enrichment Analysis (GSEA) was employed to investigate the role of genes in biology (17). Analyses using KEGG, GO, and GSEA were performed using the R package “clusterProfiler” (version 3.14.3). Significantly enriched biological processes, molecular functions, cellular components, and pathways were chosen based on a threshold of *p*-value < 0.05 and an false discovery rate (FDR) < 0.05. The STRING database (https://cn.string-db.org/) was used for the protein–protein interaction (PPI) network analysis of DEGs related to asthma. It provides uniquely comprehensive coverage, integration, and interactions obtained through text mining (18).

### 2.4 Techniques for machine learning to find diagnostic indicators

Using the training set, we applied two types of machine learning algorithms to predict asthma disease-associated genes: SVM-RFE and LASSO regression models. The LASSO regression is a model for variable selection and complexity regularisation. We used the “Venn” package to intersect the diagnostic markers of asthma patients generated by the LASSO regression and the techniques of SVM-RFE. To gauge the performance of these models, we deployed the ROC curve and computed the AUC. The AUC quantifies the capacity of these two models to discriminate between healthy control and asthma samples, with predictions based on the chosen features. In summary, we implemented the LASSO regression and SVM-RFE techniques in the training set to select and utilise the training and test sets to then identify important DEGs for asthma diagnosis and evaluate the models' performance by employing the ROC curve and AUC values.

### 2.5 Connection between immune cells and core genes

We performed immunoinfiltration analysis using the “CIBERSORT.R” package in the training cohort, used the “corrplot” packages to analyze the differentiated infiltration of immune cells between healthy people and patients with asthma, used the “preprocessCore” package to generate immunocyte content, and analyzed the correlation of core gene expression and immune cells by the “reshape2”, “ggpubr”, and “ggExtra” packages. Data with *p*-values < 0.05 and *q*-values < 0.05 were retained and volplot, barboplot, lollipop pictures were generated.

### 2.6 Animal experiments

Female wild-type C57BL/6 mice were obtained from the Beijing Vital River Laboratory Animal Technology Co (Beijing, China). Mice were provided unlimited access to water and standard food and were raised under specific pathogen-free conditions (22°C ± 1°C, 50% ± 5% humidity) with a light/dark cycle of 12 h/12 h. One week before use, the mice were isolated and acclimated. Ten mice were classified into two groups (*n* = 5 each): sham and ovalbumin (OVA). Mice in the OVA group were given an intraperitoneal injection of sensitized OVA (100 μg; Cat #: A5503, Sigma-Aldrich, USA) and aluminium hydroxide (1 mg; Cat #:77161, Thermo Fisher Scientific, USA) on days 1 and 13, while saline (100 μl) was injected into mice in the Sham group. Mice in the OVA group were administered 2% OVA in an aerosol form for over 30 min for seven consecutive days, while mice in the sham group were administered saline. All animals were sacrificed within 24 h of the last nebulization. The Ethics Committee of the Wenzhou Medical University Laboratory Animal Resource Center (Wenzhou, China) granted consent for all studies to be conducted in accordance with the ARRIVE criteria.

### 2.7 Transcriptome analysis

Library building for the high-throughput sequencing of the eukaryotic reference transcriptome from the examined mouse lung tissue samples was performed by LC-Bio Technology Co., Ltd. (Hangzhou, China). An Illumina HiSeq X Reagent Kit (Illumina, San Diego, USA) was used as the sequencing platform. The expression of each transcript was calculated using the fragments per kilobase per million read technique to identify the DEGs between samples.

### 2.8 Statistical analysis

For statistical analysis and visualisation, R version 4.2.2 was employed, while GraphPad Prism 8.2.0 was used to statistically analyze the images. Student's *t*-tests were used to compare group variables. Results were shown as the mean ± standard deviation. At least three replicates were performed for each experiment. Statistical significance was set at *p* < 0.05.

## 3 Results

### 3.1 Machine learning model development and identification of critical genes for asthma discrimination

**Figure 1:**
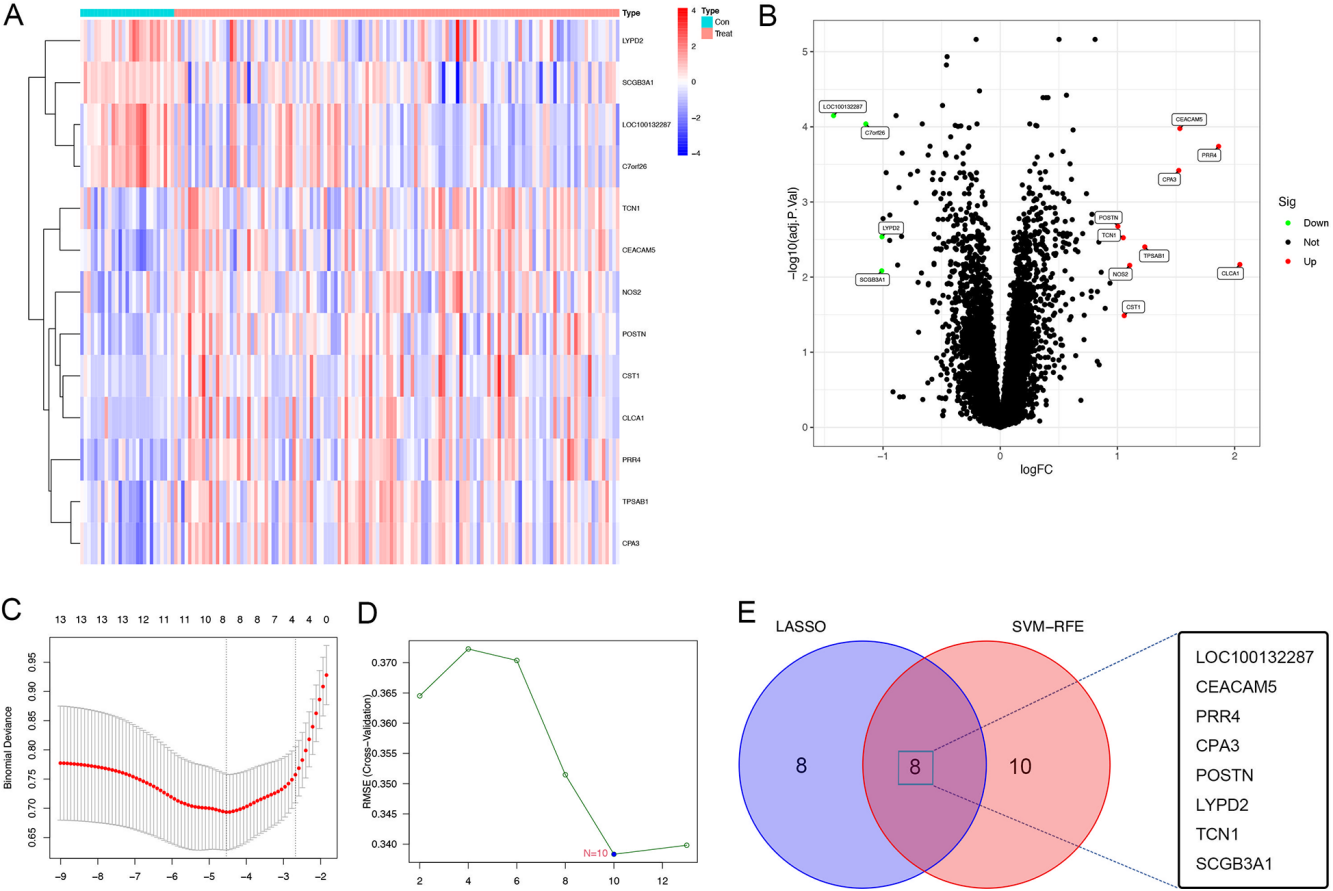
DEGs of patients with asthma using the GSE63142 datasets. **(A)** Heatmap of the the GSE63142 datasets; gene upregulation is indicated by red and gene downregulation is indicated by blue,highlighting the differences in gene expression between healthy controls and asthma patients. **(B)** Volcano plot of the GSE63142 datasets; significant DEGs with a conservative threshold of |log2FC| > 1.0, *p* < 0.05; red represents gene upregulation and blue represents gene downregulation. 13 genes were differentially expressed between healthy controls and patients with asthma (9 genes upregulated and 4 genes downregulated). **(C)** The LASSO regression analysis to identify the most relevant genes for asthma diagnosis based on the differential expression analysis results, which identified 8 diagnostic core genes. **(D)** The SVM-RFE menthod using the e1071, kernlab and caret package, which identified 10 diagnostic core genes. **(E)** Venn plot depicting the identification of key genes for distinguishing between healthy and asthma patients. The intersection of two machine learning algorithms—lasso regression and SVM-RFE method—reveals 8 pivotal genes (*LOC100132287*, *CEACAM5*, *PRR4*, *CPA3*, *POSTN*, *LYPD2*, *TCN1*, and *SCGB3A1*) as robust and discriminative features. DEGs, differentially expressed genes; LASSO, least absolute shrinkage and selection operator; SVM-RFE, support vector machine recursive feature elimination.

A total of 30,889 genes from 155 bronchial epithelial cell samples obtained from 128 patients with asthma and 27 healthy individuals were included in the training set from the GEO dataset. The age range of subjects was 18–62 years, with an average age of 37 years. We first divided the data into two groups to examine the gene expression matrix of the training set: healthy controls and patients with asthma. The subsequent DEG analysis revealed a notable variation in gene expression between the two groups, as demonstrated in the heatmap, highlighting the distinct gene expression landscape of patients with asthma (Figure 1A). A comparatively small number of DEGs were found in this comparison, with a volcano plot displaying 9 upregulated and 4 downregulated genes with a conserved threshold of |log2FC| > 1.0, *p* < 0.05 (Figure 1B).

We constructed the LASSO regression model for feature selection, which identified 8 diagnostic core genes of asthma. This approach reduced unimportant feature coefficients to zero. The SVM-RFE is a learning algorithm used in nonlinear classification, which constructs a hyperplane in the feature classes with a maximum margin (19, 20). The SVM-RFE method is used to identify the most critical diagnostic markers associated with asthma progression. Using the SVM-RFE approach, 10 asthma diagnostic genes were identified. Through the convergence of the SVM-RFE approach and LASSO regression, eight important genes were found to be discriminative characteristics that might be used to separate asthma sufferers from healthy individuals [*LOC100132287*, carcinoembryonic antigen-related cell adhesion molecule 5 (*CEACAM5*); proline-rich 4 (*PRR4*); carboxypeptidase A3 (*CPA3*); periostin (*POSTN*); LY6/PLAUR domain cintaining 2 (*LYPD2*); transcobalamin 1 (*TCN1*); and secretoglobin family 3A member 1 (*SCGB3A1*)], as shown in the Venn diagram (Figures 1C–E).

### 3.2 Pathway enrichment analysis

**Figure 2:**
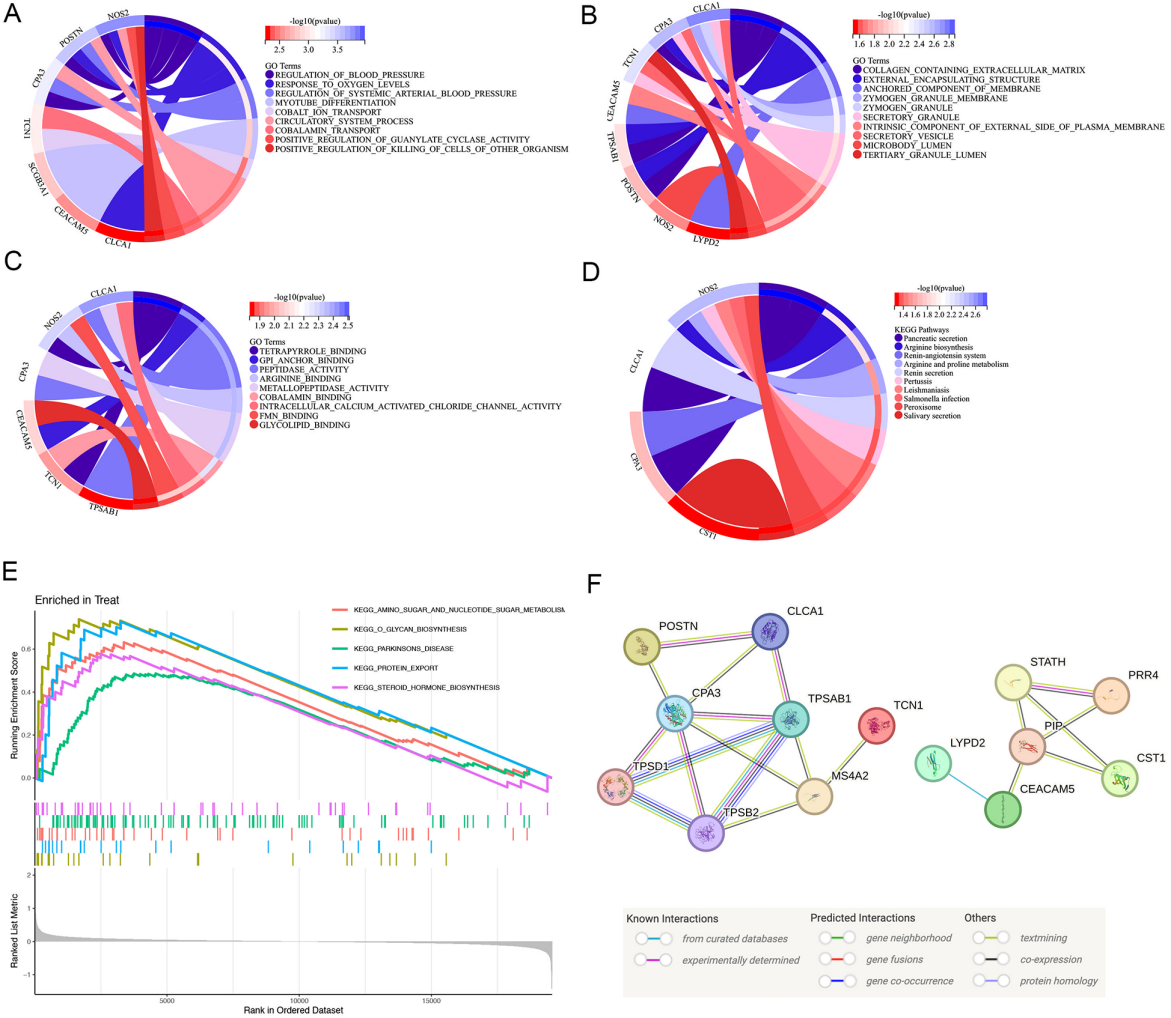
Function, pathway of the DEG enrichment analysis. **(A–C)** GO analysis of DEGs. Enrichment analysis of DEGs, demonstrating a strong association with response to oxygen levels and positive regulation of guanylate cyclase activity in BP, collagen-containing extracellular matrix, zymogen granule and anchoring membrane component in CC, and gpi anchor binding, peptidase activity, arginine binding and intracellular calcium activated chloride channel activity regard to MF. **(D)** KEGG pathway analysis of DEGs. The DEGs were linked to the pancreatic secretion, arginine biosynthesis, renin-angiotensin system and arginine and proline metabolism using KEGG pathway analysis. **(E)** Annotated KEGG pathway analysis using the GSEA approach. The results highlighted significant enrichment of amino sugar and nucleotide sugar metabolism, glycan biosynthesis, parkinsons disease, protein export, steroid hormone biosynthesis. **(F)** Interaction network of the proteins regulated by the key genes. PPI network of 13 specific asthma genes reflected the core connectivity of CPA3 and TPSAB1 in these proteins. DEGs, differentially expressed genes; GO, gene ontology; KEGG, Kyoto encyclopedia of genes and genomes; PPI, protein–protein interaction; GSEA, gene set enrichment analysis.

Considering the significant variations in asthma gene expression patterns, we continued with a more comprehensive study focusing on the 13 DEGs. According to the results of the GO enrichment analysis, 95 GO items comprised 19 molecular functions (MF), 64 biological processes (BP), and 12 cellular components (CC). Several processes were significantly enriched. Notably, these included the response to oxygen levels and positive regulation of guanylate cyclase activity for BP, collagen-containing extracellular matrix, zymogen granule and anchoring membrane component for CC, and gpi anchor binding, peptidase activity, arginine binding and intracellular calcium activated chloride channel activity regard to MF. These findings underscore the importance of protein metabolism and enzyme regulation in the pathogenesis of asthma (Figures 2A–C).

We performed the KEGG pathway analysis to explore the higher levels of biological functions associated with asthma. This approach focuses on how a collection of genes in the genome is linked to gene products (enzymes) to create pathways that are distinct in any particular organism (21, 22). According to the KEGG pathway analysis, the DEGs were primarily linked to the pancreatic secretion, arginine biosynthesis, renin-angiotensin system and arginine and proline metabolism (Figure 2D). Nitric oxide is a vasodilator with anti-inflammatory and bronchodilatory properties. Since arginine stimulates the synthesis of nitric oxide, it is possible that changes in the arginine metabolome contribute to the pathophysiology of asthma (23). KEGG enrichment analysis further emphasized that the DEGs were deeply intertwined with the pathogenesis of asthma.

Based on the findings of the KEGG enrichment study, we annotated KEGG using the GSEA approach. The results highlighted significant enrichment of amino sugar and nucleotide sugar metabolism, glycan biosynthesis, parkinsons disease, protein export, steroid hormone biosynthesis. There was significant enrichment of protein export and amino sugar and nucleotide sugar metabolism. Thus, protein, amino sugar and nucleotide sugar are crucial for the pathophysiology of asthma (Figure 2E).

### 3.3 PPI network analysis of asthma related DEGs

To create a PPI network, thirteen primary DEGs associated with asthma were imported, examined, and visualized using SVG in the STING database. The total gene scores are shown as the number and colour of lines connecting the nodes. Fifteen proteins combined with CPA3 and 19 proteins combined with TPSAB1, reflecting the core connectivity of CPA3 and TPSAB1 (Tryptase alpha/beta-1) in these proteins (Figure 2F). Among these, POSTN, CLCA1 (Calcium-activated chloride channel regulator 1), CPA3, and TPSAB2 (Tryptase alpha/beta-2) existed some co-expression possibility.

### 3.4 Validation of the external dataset's main genes

**Figure 3:**
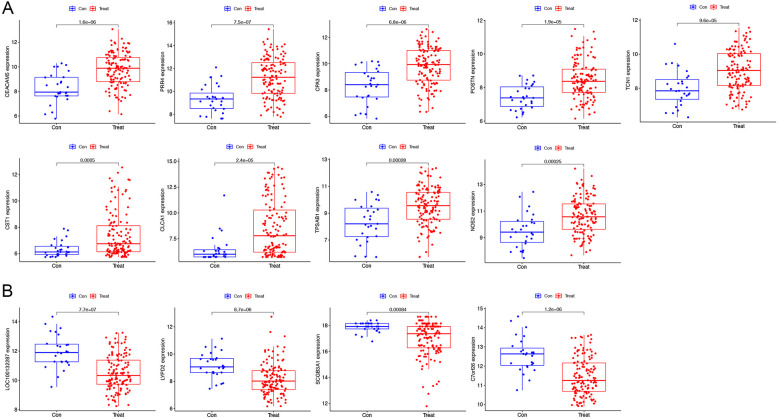
Verification of the varying expression of diagnostic genes. The differential expression of the GSE63142 dataset. **(A)** The genes have high levels of expression in patients with asthma. **(B)** The genes have low levels of expression in patients with asthma.

Our findings from the GSE63142 dataset showed 13 genes differing in asthma. We further used a one-to-one format for comparison and validated with the GSE63142 dataset The genes *CEACAM5*, *PRR4*, *CPA3*, *POSTN*, *TCN1*, *CST1* (Cystatin-SN), *CLCA1*, *TPSAB1* and *NOS2* (Nitric oxide synthase 2) were highly expressed in patients with asthma (Figure 3A). *LOC100132287*, *LYPD2*, *SCGB3A1* and *C7orf26* (Chromosome 7 open reading frame 26) had low expression in asthma patients (Figure 3B). The validity of these gene transcription patterns as diagnostic markers for asthma was supported by the consistency of the genome data. Further research is required to fully elucidate the underlying molecular pathways. *LOC100132287* gene did not present in GSE158752 dataset. *PRR4*, *TCN1*, *CST1*, *CLCA1*, and *NOS2* were also highly expressed in patients with asthma in GSE158752 dataset (Supplementary Figure S1A). *LYPD2* and *C7orf26* were also lowly expressed in patients with asthma in GSE158752 dataset (Supplementary Figure S1B).

### 3.5 Analysis of diagnostic indicators using ROC curves

**Figure 4:**
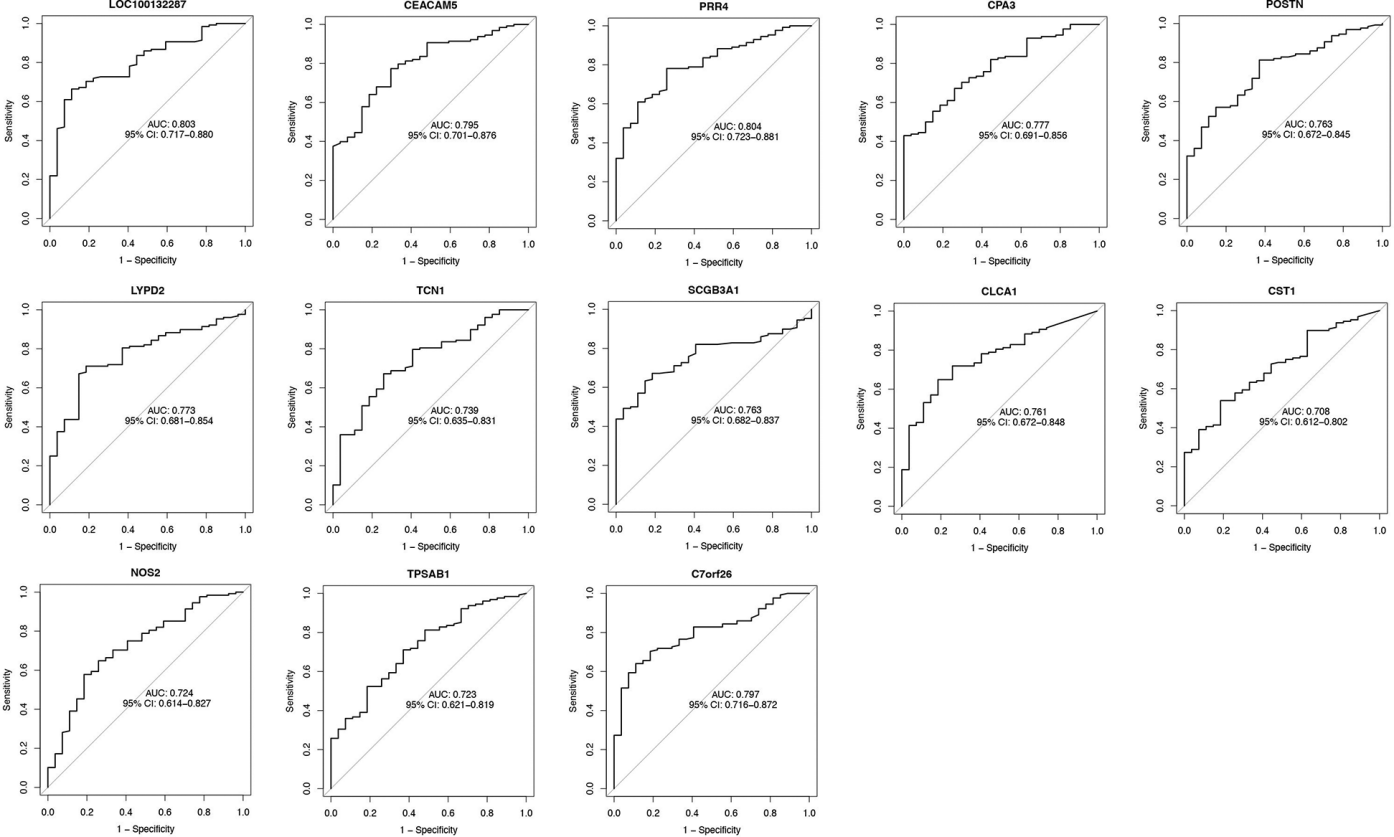
The AUC of diagnostic genes of the GSE63142 dataset. We used the ROC curve and calculated AUC to assess the performance of these models. The AUC ranged from 70.8% to 80.4%. ROC, receiver operating characteristic; AUC, area under the receiver operating characteristic curve.

Using the AUC of the ROC curve, we verified the performance of the 13 asthma-associated genes in the training set and GSE63142 dataset. We created an ROC curve drawing of the diagnostic markers in RStudio to determine their diagnostic utility. The AUC ranged from 70.8% to 80.4% in the training set (Figure 4). The AUC values showed that the GSE63142 dataset performed satisfactorily overall, which suggests that these thirteen genes contributed significantly to the diagnostic utility of disease classification. Greater accuracy in differentiating between healthy individuals and asthma sufferers was indicated by the higher AUC values. The AUC ranged from 54% to 83% in the test set. *CLCA1* reached an AUC value of 0.83 and its predictive efficacy was even higher than that of the training set (Supplementary Figure S2).

### 3.6 Differentiation of immune characteristics and the immune cell correlation analysis

**Figure 5:**
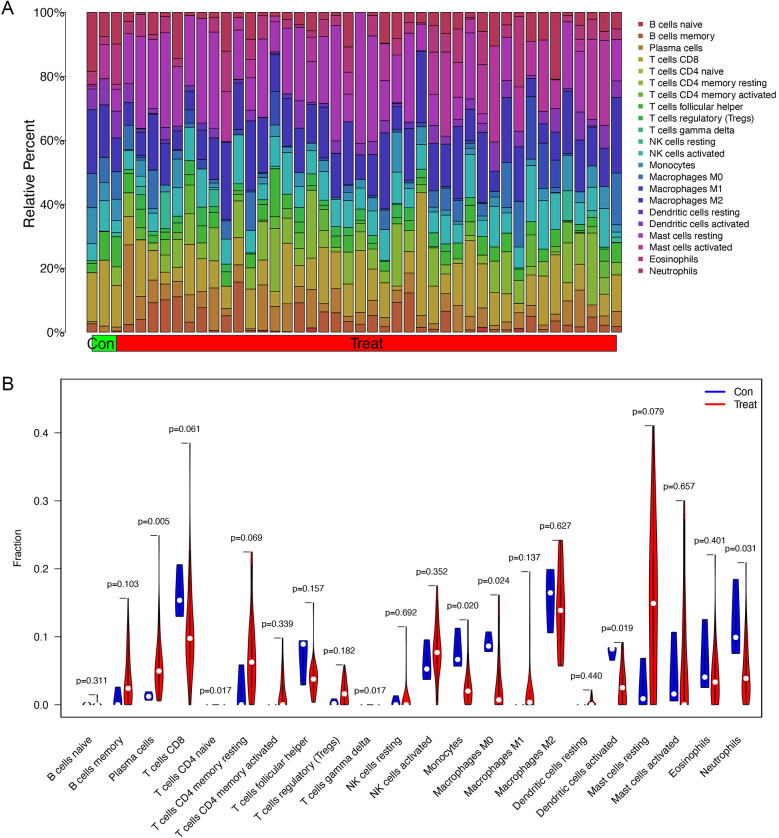
Immune cell infiltration analysis between asthma and healthy subjects. **(A)** The proportion of immune cells in each sample. **(B)** Differential infiltration of immune cells between healthy controls and asthma patients. Plasma cells, T cell CD4 naive, T cell gamma delta, monocytes, macrophages M0, activated dendritic cells and neutrophils have significant differences between two groups.

**Figure 6:**
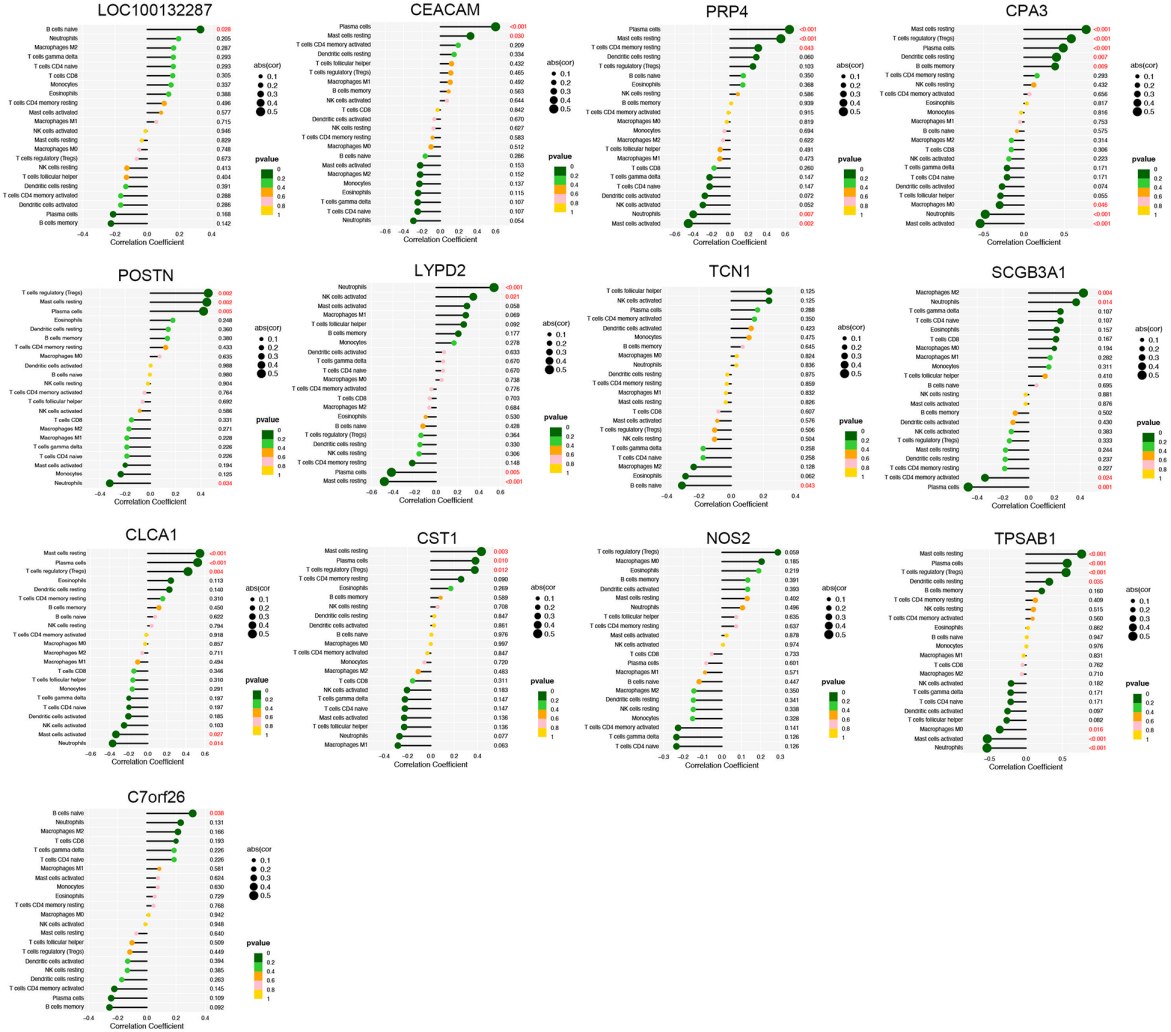
Immune cell correlation analysis of diagnostic genes. The genes *CEACAM5*, *PRR4*, *CPA3*, *POSTN*, *LYPD2*, *CLCA1*, and *CST1* were significantly associated with plasma cells and resting mast cells (*p* < 0.05).

The proportion of immune cells in each sample was obtained by immune cell infiltration analysis. plasma cells, T cell CD4 naive, T cell gamma delta, monocytes, macrophages M0, activated dendritic cells and Neutrophils were differential between healthy controls and asthma patients (Figures 5A,B). The findings of our investigation into the relationship between DEGs and immune cells are displayed in lollipop charts (Figure 6). The genes *CEACAM5*, *PRR4*, *CPA3*, *POSTN*, *LYPD2*, *CLCA1*, and *CST1* were significantly associated with plasma cells and resting mast cells. The genes *LOC100132287*, *TCN1,* and *C7orf26* were significantly associated with naive B cells. The genes *SCGB3A1* were significantly associated with plasma cells, neutrophils and active memory CD4 T cells. The genes *CEACAM5*, *PRR4*, *CPA3*, *POSTN*, *CLCA1*, and *CST1* had positive correlations with plasma cells and resting mast cells (*p* < 0.05), whereas *PRR4* and *CPA3* had negatively correlated with neutrophils and active mast cells (*p* < 0.05). The genes *CPA3*, *POSTN*, *CLCA1*, *CST1*, and *TPSAB1* were significantly associated with regulatory T cells Tregs (*p* < 0.05).

### 3.7 Genetic validation in an asthmatic mouse model

**Figure 7:**
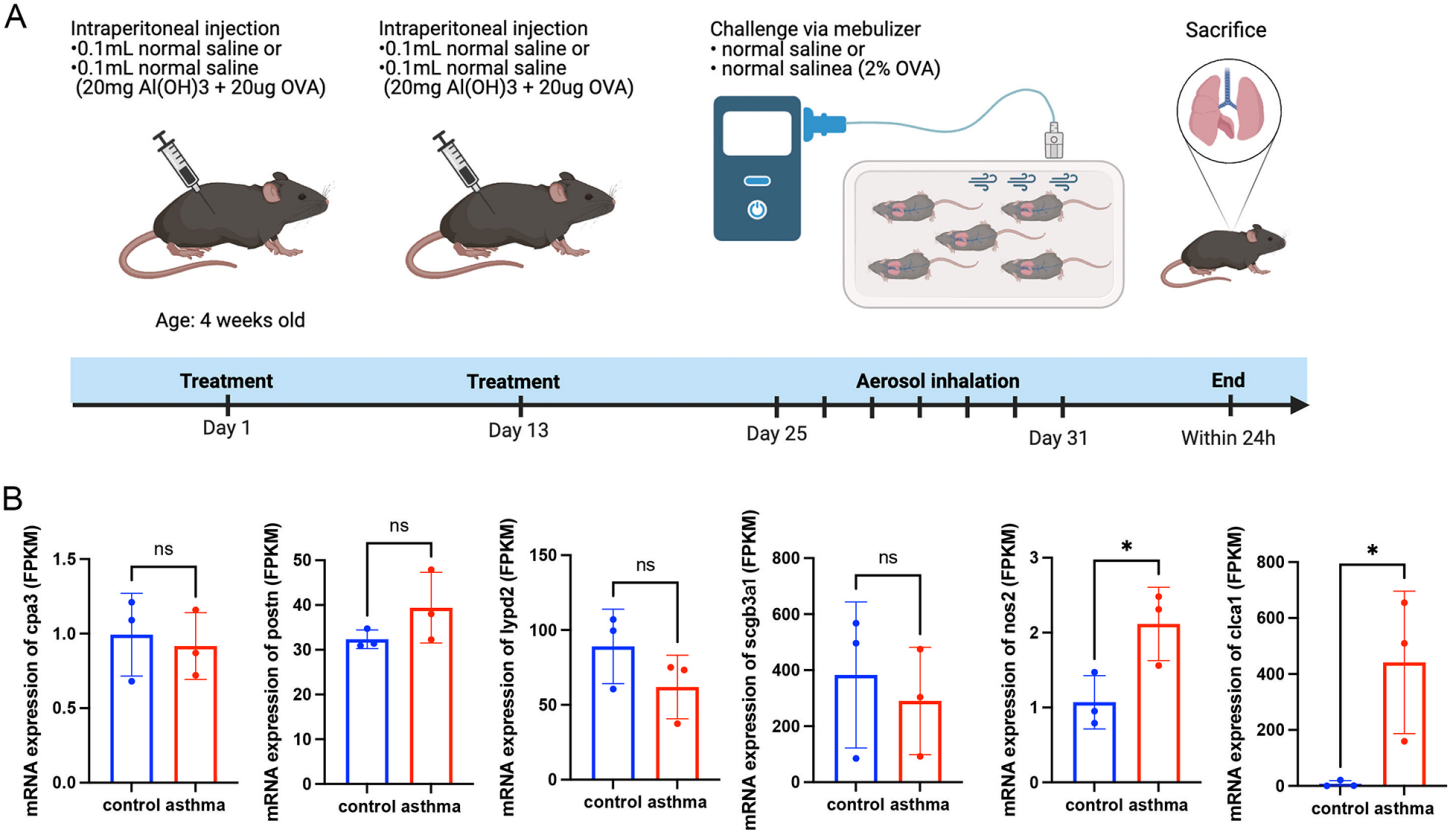
Animal experiments and sequencing analysis. **(A)** Schematic of the OVA asthma model construction; **(B)** Gene expression levels in mouse lung tissue. Control: normal mice; Asthma: asthma model mice. OVA, ovalbumin. Transcript analysis of the gene mRNA expression levels of *cpa3, lypd2, scgb3a1, nos2*, and *clca1* in murine lung tissues, showing the gene nos2 and clca1 expression trends consistent with the transcriptomic data in patients with asthma.

To determine gene expression levels, we extracted RNA from mouse lung tissues and performed high-throughput eukaryotic sequencing analysis. These four murine homologous genes, *cpa3, postn, lypd2*, and *scgb3a1*, did not differ significantly between the asthma group and the normal group. However, these findings indicated that the mRNA expression of *nos2* and *clca1* in the asthma group was higher than that in the normal group, which was in line with the transcriptome observations made in asthmatic patients in Figures 1A,B (Figure 7).

## 4 Discussion

Asthma, an prevailing pulmonary malaise, afflicts many individuals across the world (24). Wheezing, shortness of breath, coughing, and tightness in the chest are some of the symptoms that are caused by inflammation and constriction of the airways (25). Currently, we know several pathogenetic mechanisms contribute to the development and progression of asthma: inflammation, airway hyperresponsiveness, airway remodelling, immunological factors, genetic predisposition, environmental factors, and neural control (26). Many pharmaceutical strategies and self-management methods have been developed. However, some patients with asthma cannot control their symptoms with available treatment and management strategies, which highlights the unmet medical needs of these patients, an incomplete understanding of asthma pathogenesis, and the need for continued exploration of the topic, such as by targeting the specific genes that play cardinal roles in asthma evolution and progression. In this context, machine learning and other advanced techniques can be used to identify undiscovered genes critical to asthma and further explore asthma pathogenesis.

To address knowledge gaps physiology and pathology of asthma, we used two machine learning models to determine the main genes responsible for the initiation and progression of asthma. Preliminary exploration revealed marked disparities in gene expression between patients with asthma patients vs. that in healthy controls. We utilized KEGG, GO, GSEA, and PPI network analyses to further confirm the correlation of differential gene expression and asthma. Merging the LASSO regression with the SVM-RFE method enabled a model that not only showed a consensus in identifying key asthma genes but also compensated for performance deficiencies of the individual models in terms of accuracy and prediction. This synthetic approach identified eight pivotal asthma-related genes spanning the training set, test cohorts, and experimental animal specimens.

We identified 8 key genes (*LOC100132287*, *CEACAM5*, *PRR4*, *CPA3*, *POSTN*, *LYPD2*, *TCN1*, and *SCGB3A1*) associated with asthma by combining the LASSO regression model and the SVM-RFE method. Higher AUC values indicated a high diagnostic value. The association of the five genes *CEACAM5*, *POSTN*, *TCN1*, *SCGB3A1,* and *CPA3* with asthma has been extensively identified and validated, and these are the most highly upregulated genes in patients with asthma (27–31), and *CEACAM5* is associated with resting mast cells and eosinophils (32). The genes *POSTN*, *TCN1*, and *CPA3* are associated with the type 2 inflammatory response (26, 33, 34). The gene *SCGB3A1* is highly expressed in sputum columnar cells in patients with severe asthma and associated with non-neutrophilic airway inflammation (35). This is consistent with our findings.

Current reports indicate that *PRR4* may impact the efficiency of the submucosal glands, leading to pathological changes in the respiratory tract (36). The role of the gene *PRR4* in asthma require further verification. To date, no study has demonstrated an association between asthma and *LYPD2*. Some studies have shown that LYPD2 is predicted to be a GPI-anchored Ly6 protein, enriches in non-classical monocytes (37, 38). However, the gene *LYPD2* had high AUC values, indicating that it has good predictive efficacy, which requires further exploration of the relationship with asthma.

The current study had several limitations. The foundation of our investigation was the computational analysis of gene expression samples, which provided estimated results and reflected our reliance on computational data. Not all core genes were validated in asthmatic mice, partly because some genes are only significantly expressed in subjects with severe asthma, relatively small sample size of the mouse experiments and not all genes are homologous in humans and mice. In future studies, we will continue to examine the association between the newly discovered diagnostic genes and asthma. Meanwhile, we are continuing to confirm the efficiency of these genes in the diagnosis of asthma in clinical applications.

In conclusion, our study validated several key genes potentially associated with asthma (*CEACAM5*, *PRR4*, *CPA3*, *POSTN*, *TCN1*, and *SCGB3A1*) and identified new asthma genetic marker, such as *LYPD2*. We propose that the combination of the detection of these genes and patient symptoms can lead to the prediction and diagnosis of asthma. We hope that by detecting the proteins expressed by these genes, we can determine the therapeutic effect of asthma and achieve clinical management.

## Data Availability

The GSE63142 and GSE158752 asthma datasets were obtained from the GEO database at the National Center for Biotechnology Information (NCBI) (https://www.ncbi.nlm.nih.gov/geo/).

## Supplementary material

Supplementary Figure S1 The differential expression of the GSE158752 dataset. **(A)** The genes have high levels of expression in patients with asthma. **(B)** The genes have low levels of expression in patients with asthma.

Supplementary Figure S2 The AUC of the GSE158752 dataset. Validation of the 13 key genes using ROC curves in the test set, which ranged from 54% to 83%. AUC, area under the receiver operating characteristic curve.
